# Haberlandt’s dream and the secrets of the kitchen

**DOI:** 10.1007/s00709-026-02174-1

**Published:** 2026-02-20

**Authors:** Peter Nick

**Affiliations:** Joseph Gottlieb Kölreuter Institute für Plant Sciences, Karlsruhe Institute of Technology, Karlsruhe, Germany

When zoologist Theodor Schwann and botanist Matthias Schleiden coined the Cell Theory (for plants: Schleiden 1838; as general concept: Schwann 1839), their boldest claim (among many bold claims) was that cells represent elementary organisms, principally able to generate the entire life form. Half a century later, this implication launched an important research programme. Haberlandt (1902) proposed that culture of plant cells should not only allow for interesting insight into the properties and potentialities as elementary organisms, but would also reveal the interactions and influences they experience when integrated into multicellular entities. He also described his attempts to cultivate cells from different sources in simple nutrient solutions. Although he succeeded to keep sterility, the cells did not divide, but just expanded, because the hormones needed to stimulate cell division had not been discovered yet. Another half of a century had to elapse, until the first plant tissue cultures became available (Muir et al. 1954), which paved the way for the development of green genetic engineering from the 1980ies. In parallel, Harrison developed animal tissue culture as approach to dissect morphogenetic mechanisms isolated from the complexity of the living organism (Harrison 1912). Also, his experiments failed in the beginning. When he tried to cultivate neural tissue originating from frog embryos in a hanging drop, they simply died. He was luckier than Haberlandt, recognising the need of a solid substrate. First experiments with gelatine did not help, but clotting lymph from adult frogs, a source rich in fibrinogen, was successful, a breakthrough enabling rapid progress in the understanding of neural development.

Thus, already from its beginning, the history of tissue culture was shaped by experimental details that, at their time, were not predicted by conceptual insight, but rather discovered by serendipity and the right intuition. Two contributions to the current issue, one from the plant, and one from the animal field, illustrate that the right recipe has remained a crucial factor even in our days.

The study by Li et al. (2026) develops, very systematically, a method to express genes of interest in protoplast from *Rhododendron* petals. This origin of this genus is in China, where there exist more than 500 species that are valued for the beauty of their flowers and have been used for horticulture all over the world. Although the genomes of several species have been established, the functional annotation has remained limited, because transformation and callus regeneration is cumbersome and lengthy for this woody plant. There is considerable interest, however, to establish functional genomics for the purpose of breeding of new ornamental forms or improved climate resilience. The scope of the authors was, therefore, to develop a highly efficient transient expression system in *Rhododendron*. They decide to go for a protoplast-based protocol, because this allows for high transformation efficiency. However, the bottleneck here is the quality, yield, and uniformity of the protoplasts. Composition and thickness of the cell wall are crucial factors. They decide to use a very unusual source material – the delicate petals, because the walls of these cells are thin, favouring the release of the wall-free protoplasts. As additional turn, they peel of the epidermis, improving the penetration of wall-digesting enzymes. By comparing a panel of enzyme combinations, hydrolytic conditions, and osmotic parameters (as wall-free cells, the protoplasts are stable only in a more or less isotonic environment), they arrive at a high yield. However, as often in protoplasting, debris from burst cells is an issue, which is then successfully solved by centrifugation at low speed through a sucrose cushion, where the protoplasts remain afloat, while debris is precipitated. The success of the methodology is then demonstrated by visualising the subcellular localisation of two candidate proteins known as regulators of heat and cold adaptation. The efficiency of this system is sufficiently high to monitor temporal dynamics of expression for the introduced genes. Encouraged by this success, the authors propose to develop the system further towards protoplast regeneration, which would short-cut the lengthy process of conventional transformation and not only allow for more efficient gene annotation, but also for novel strategies in breeding *Rhododendron*. This study can serve as paradigm, how thorough knowledge of the principles behind a method can really help to get full control over an experimental system – a message that is the more important in times, when molecular biology is shaped by the use of ready-to-use kits, where the user often does not have a clue, how it works, and how to fix it, when it does not work.

In their review, Pristyazhnyuk et al. (2026) explain the potential and challenges of glioma cell cultures as tool to understand the factors contributing to this most common of all neural cancers. To assess the causes behind malignancy and to test the effect of potential medication, glioma cell cultures are mandatory. Commercially available glioma-derived cell lines, a common tool in the field, are not really helpful – while they are standardised, they only poorly reflect the situation in individual patients. Experimental models need to represent what they are used for, otherwise they are pointless as models. The authors make a strong point in favour of patient-derived primary cell cultures. Bio-banks from patient samples are available and would allow for very specific approaches. However, the variability of the primary cell cultures derived from such samples is high, making it hard to come to hard conclusions. Therefore, in their review, the authors walk the reader through the individual steps, describing the crucial factors, the state of the art, and the potential routes for future research. A central issue of any protocol is, to what extent it will preserve the glioma stem cells, whether the sample heterogeneity (possibly reflecting different cell types) is representative, and whether cultivation will shift this heterogeneity by selecting for specific genotypes. The screws for experimental control are the enzymes used for dissociation of the sample tissue, amount and profile of growth factors in the culture media, the topology of cultivation (monolayers versus neurospheres and organoids), the serum, coating of the vessel surface, and the details of excision, mainly with respect to the recovery of glioma stem cells, as well as the right micro-environment shaping stemness versus differentiation. The authors add also a detailed method critics, describing the drawbacks of the individual strategies, and how one can assess them. For instance, the epithelial-mesenchymal transition, a key point for tumour invasiveness, needs to be retained. Since this re-differentiation process is common in wound healing and early embryogenesis, the specific signal pathways active there can be probed to judge the experimental potential of a given primary cell culture. They propose that for a given sample, one should go for a systematic combination of standard protocols in parallel and monitor the outcome by a panel of quality controls which are also discussed.

Both contributions focus on method development and optimisation, an aspect that should not be neglected. Experiments are always reduced representations of a reality that is far more complex. By standardisation, researchers try to infer causal links and to construct working hypotheses that help to ask more precise and substantial questions. The success of hypothesis-driven research is often limited by noise in the experimental system. This is particularly true for biology dealing with systems that are highly complex, non-linear, and individual. Whether a far-reaching dream becomes true or ends in a collection of noisy data that will remain non-informative is often not decided on the level of theoretical concepts, but by the secrets of the kitchen.
